# A minimal model of PTSD susceptibility and timescales

**DOI:** 10.1016/j.isci.2026.116391

**Published:** 2026-06-24

**Authors:** Yaniv Grosskopf, Dor Danan, Avi Mayo, Keren Doenyas-Barak, Uri Alon

**Affiliations:** 1 Department of Molecular Cell Biology, Weizmann Institute of Science, Rehovot 76100, Israel; 2 Sagol Center for Hyperbaric Medicine and Research, Shamir MC, Be’er Ya’akov, Israel; 3 Tel Aviv School of Medicine, Tel-Aviv University, Tel Aviv, Israel

**Keywords:** Neuroscience, Psychiatry, Computational modelling, Systems Biology, Mathematical Modeling

## Abstract

Post-traumatic stress disorder (PTSD) is a debilitating condition characterized by intrusive memories, hyperarousal, and avoidance. Treatment outcomes vary, and mechanistic understanding remains incomplete. After trauma, only a minority (10–20%) develop chronic symptoms, while others recover over varying timescales. We present a minimal mathematical model using an autocatalytic symptom driver balanced by removal, capturing PTSD’s bistability - a persistent switch to a high-symptom state after trauma. Individual susceptibility is determined by a single “resilience” parameter R, such that PTSD occurs only at low R. The model explains the broad distribution of recovery timescales at intermediate R (dynamical ghost). It also predicts that treatment must surpass a threshold to produce lasting improvement, consistent with clinical observations. We validate the model against longitudinal symptom data across multiple cohorts and trauma types.This simplified framework links physiology to trauma response dynamics and may help guide individualized treatment.

## Highlights

• Minimal model explains PTSD bistability using a single resilience parameter
• Dynamical ghost explains the broad recovery timescales observed after trauma
• Model suggests treatment may require crossing a threshold for lasting recovery
• Model accurately fits longitudinal PTSD data across diverse cohorts

## Graphical abstract

**Figure:**
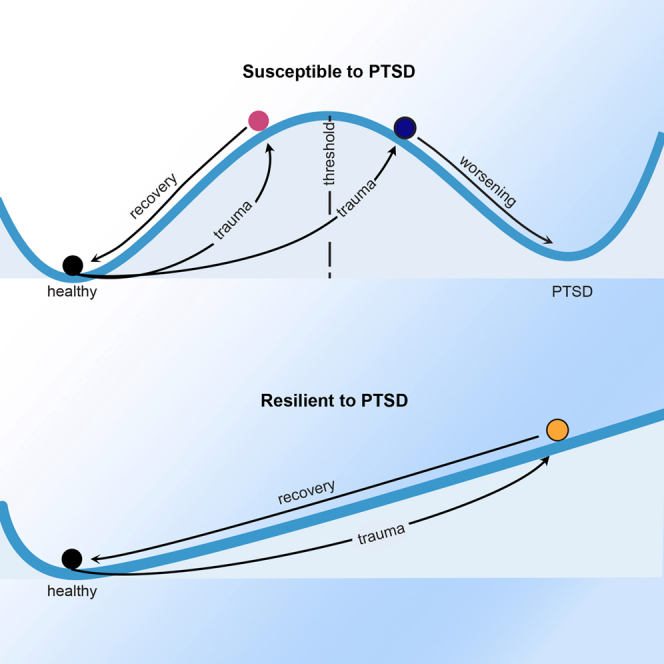

## Introduction

Post-traumatic stress disorder (PTSD) is a chronic psychiatric condition in which a traumatic event causes flashbacks, mood and memory symptoms, hypervigilance, and avoidance. PTSD can cause severe impairment in work and social functioning.^1^^,^^2^^,^^3^ Symptom severity and diagnostic status are commonly assessed using standardized instruments. The Clinician-Administered PTSD Scale (CAPS) is the gold-standard structured interview for PTSD diagnosis and severity quantification, capturing symptom frequency and intensity across re-experiencing, avoidance, negative cognitions/mood, and hyperarousal clusters.

Acute stress disorder (AcSD) is diagnosed when trauma-related symptoms are elevated during the first month post-trauma. PTSD is diagnosed if these symptoms persist beyond one month.^1^ Symptoms can also have a delayed onset, with some individuals not meeting full diagnostic criteria until several months or even years after the traumatic event.^4^

About 10% of the population is susceptible to PTSD in many types of trauma, including accidents, military, loss, and health-related trauma. Childhood trauma shows lower prevalence, and war/refugee trauma shows higher prevalence per exposure in some studies.^5^ The remaining ∼90% of the population recovers after trauma, but with a wide range of recovery times. Most individuals (∼70%) experience an acute stress response, as they develop symptoms and recover within days after the traumatic event. About 20% of the population develops longer-lasting symptoms after experiencing a traumatic event and recovers on a timescale of weeks to months, a condition known as AcSD.^1^^,^^2^

The biological mechanisms of PTSD are complex and heterogeneous, involving multiple systems, including the central nervous system, endocrine systems, and the autonomic nervous system.^3^^,^^6^^,^^7^^,^^8^^,^^9^^,^^10^ Neuroimaging studies have identified elevated amygdala activity, reduced functional connectivity in the prefrontal cortex, and decreased hippocampal volume and activity.^6^^,^^10^^,^^11^^,^^12^^,^^13^

Treatments for PTSD primarily involve trauma-focused psychotherapies, which address maladaptive fear networks and trauma-related conditions.^14^^,^^15^ Pharmacological options, such as SSRIs, are also used to reduce core symptoms such as hyperarousal, re-experiencing, and avoidance.^16^ In addition, hyperbaric oxygen therapy (HBOT) has proven to be a helpful treatment, particularly for individuals with treatment-resistant PTSD.^17^^,^^18^ However, many individuals do not fully recover with existing interventions, and we still lack a comprehensive understanding of PTSD mechanisms, reliable diagnostic methods, and strategies for tailoring interventions for each individual.^19^

A fundamental feature of PTSD is the existence of a healthy state that is disrupted after trauma, leading to entry into a high symptom PTSD state that is stable for a long time as revealed by longitudinal studies.^5^^,^^20^^,^^21^ Psychiatric literature discusses such states as attractors.^22^^,^^23^ Mathematically, the feature of having two stable states is called bistability, as has been previously applied in major depression.^24^ Bistability is the coexistence of two stable states, a “healthy” or low-symptoms state, and a “PTSD” or high-symptoms state. The high-symptom state can vary in severity among different individuals. This bistability is revealed in susceptible individuals when a traumatic event “pushes” them from a low-symptom but vulnerable healthy state to a high-symptom PTSD state. In contrast, non-susceptible individuals show only one, healthy, stable state. After exposure to a traumatic event, these individuals show transient symptoms, but then recover and return to their stable state.

An additional feature is that recovery spans a broad spectrum of timescales, ranging from days in resilient individuals to weeks or months in AcSD. Treatment of PTSD typically requires at least several months, and while many patients achieve sustained improvement, some experience a relapse of symptoms afterward.^5^^,^^25^^,^^26^^,^^27^

The mechanisms underlying this bistability, the broad distribution of timescales, and the variance in treatment response remain unclear. To better understand these core dynamics, we propose a minimal mathematical model that offers a top-down, timescale-based analysis of symptoms.^28^ Symptoms are represented by an autocatalytic variable *x* balanced by a linear removal. Susceptibility to PTSD is determined by a resilience parameter, *R*, that reflects individual differences in symptom dynamics shaped by genetics and early life experiences. When *R* approaches its critical threshold, the model predicts a dynamical ghost effect, accounting for prolonged recovery. It also forecasts a treatment threshold: interventions that reduce symptoms below a threshold cause lasting remission, whereas lesser improvements lead to relapse, consistent with recent HBOT findings.^29^ Finally, we discuss the biological correlates of the model parameters.

## Results

### A bistable mathematical model for PTSD

To develop a simple model with a minimal number of parameters, we consider how physiological stress circuitry might produce bistability, susceptibility, and the broad timescale spectrum of trauma response. The variable in the model is symptom load *x*. We assume a linear mapping between *x* and clinical scores such as CAPS. We remain agnostic to *x*’s physiological origin - a plausible biological correlate of *x* may arise from amygdala-hippocampus interactions (see discussion). Later, when comparing data, we convert *x* to symptom scale scores (such as CAPS scores) by multiplying *x* by a scale factor.

To describe the dynamics of *x*, we use physiological equations inspired by models of endocrine and neuronal circuits.^28^^,^^30^^,^^31^ The rate of change of *x* is a balance of symptom generation and homeostatic forces that reduce symptoms (production and removal terms). The model is thus of the form $\frac{dx}{dt}=production\left(x\right)-removal\left(x\right)$.

The following choice of production and removal is simple to analyze (other choices give similar results, see SI1).

(Equation 1) $$\frac{dx}{dt}=ax^{2}\left(1-\frac{x}{c}\right)-bx$$

The first term *ax*^2^, is self-enhancing production, in which symptoms increase their own production. To prevent symptoms from going to infinity, there is a carrying capacity term that stops production when maximal symptoms are reached *x* = *c*. The removal term −*bx* has a removal rate parameter b in units of 1/time, which allows mild symptoms to recover to zero with a half-life of *ln* 2/*b*. The cubic polynomial form of this equation is the minimal polynomial that allows two distinct stable states.

This equation has three parameters: autocatalysis (*a*), removal (*b*), and carrying capacity (*c*). We can reduce these parameters to a single effective parameter by rescaling *x*, *x* = *x*/*c*, and time *τ* = *t*·*a*·*c*. This reduces the number of parameters from 3 to a single non-dimensional parameter *R* = *b*/*ac*, which is the ratio of the removal parameter to production and carrying capacity parameters (“anti-symptom” to “pro-symptom” parameter ratio). Thus, the scaled equation for symptom load *x* has a single “resilience” parameter *R*:

(Equation 2) $$\frac{dx}{d\;\tau }=x^{2}\left(1-x\right)-Rx$$

**Figure 1:**
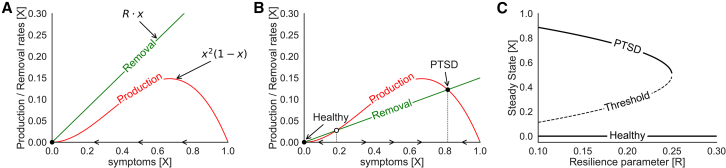
The model can show only a healthy stable point, or bistability with a PTSD high symptom state, governed by the ratio of production and removal (A) When the resilience parameter is above a threshold, *R* > 1/4, removal exceeds production, and there is only one fixed point at zero symptoms (black circles denote stable steady states), corresponding to health. (B) When *R* < 1/4, there are three fixed points-a stable fixed point at zero symptoms, a stable fixed point of high symptoms, and a middle unstable fixed point (white circle). (C) The fixed points in the model depend on the resilience parameter *R*. Below *R* = 1/4, there are two stable and one unstable fixed point. There is a stable fixed point at *x* = 0 for all values of R.

One can graphically analyze this equation with a rate plot, by plotting production and removal rates as a function of *x* (Figure 1A).^28^ The rate plot shows whether a given level of *x* (symptom load) increases or decreases: If the production curve is higher than the removal curve, *x* rises; if the removal is higher, *x* decreases. Where the two curves cross, we have a fixed point, a steady state of the system, since production equals removal and the rate of change is zero.

The model has two regimes according to the value of the parameter *R*. If *R* is larger than a critical value *R* > 1/4, the production curve is always higher than removal, except at *x* = 0, where the two curves cross (Figure 1A). Thus, there is a single fixed point at low symptoms, *x* = 0. It is a stable fixed point: *x* always declines back to zero because removal exceeds production. This regime corresponds to resilience.

In contrast, if the parameter *R* is smaller than the critical value *R* < 1/4, the production and removal curves cross three times (Figure 1B). There are three fixed points, a low-symptom healthy state and a high-symptom PTSD state, separated by an unstable fixed point.

This middle unstable fixed point x_*u*_ is a crucial threshold for those susceptible to PTSD. If a mild stressful stimulus causes *x* to rise but not exceed x_u_, *x* drops back to zero since the removal curve is higher than the production curve. However, if trauma raises x above x_u_, *x* increases and reaches the PTSD fixed point of high symptoms. This high fixed point is stable - *x* remains high even after the trigger is removed. If perturbed slightly, x returns to the high fixed point.

Thus, when trauma causes symptoms to exceed the unstable fixed point, the variable x rises and shifts to the high symptom fixed point and remains there indefinitely. Recovery is only possible if the variable *x* is brought back below x_u_, allowing the system to return to the healthy fixed point at *x* = 0.

The severity of the PTSD fixed point also depends on *R.* The lower the resilience parameter R, the higher the symptom load described by *x* (Figure 1C, top curve). In methods, we describe a linear mapping between *x* and measures such as CAPS scores for PTSD, with an individual weight factor *w*_*i*_ that takes into account individual differences not accounted for in the model.

We conclude that the model shows bistability when *R* is small, and shows a monostable healthy state when *R* is large. Susceptible individuals for PTSD are those with *R* ≤ 1/4. Thus, if parameter *R* varies in the population due to genetic factors and early life events, some individuals will be susceptible, while others will be resilient. In the model, PTSD is reversible if treatment can reduce x below the unstable threshold.

### A wide range of post-trauma recovery timescales can be explained by a dynamic ghost

We next explore the timescales of recovery. As presented above, we assume that the parameter *R*, the ratio of removal and production parameters, varies in the population. The various values of *R* represent a spectrum ranging from resilience to PTSD.

**Figure 2:**
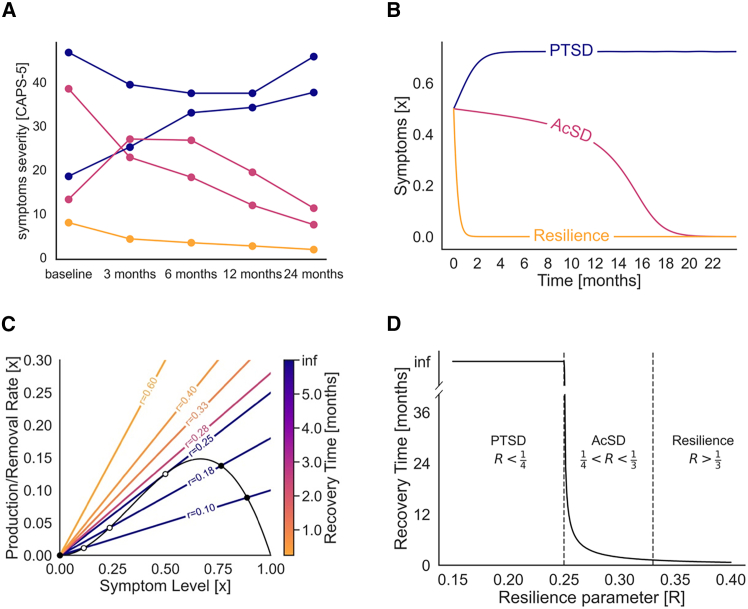
Trajectories of symptom progression in different resilience regimes (A) Data replotted from Kim et al.^25^ for PTSD symptoms as measured by the CAPS scale for individuals after a traumatic event show persistent symptoms (PTSD, blue), recovery on long timescales (ACSD, pink), and rapid recovery (orange). (B) Symptom trajectories over time in the model exhibiting resilience (orange, *R* = 0.4, acute stress disorder (ACSD, pink, *R* = 0.252), and post-traumatic stress disorder (PTSD, blue, *R* = 0.2). (C) Rate plot of production and removal for different values of the resilience parameter *R*, color-coded by recovery time. Brighter colors indicate faster recovery, while darker colors correspond to slower recovery, or no recovery at all. Filled circles denote stable fixed points, whereas open circles represent unstable ones. The dynamical ghost occurs when removal is above and almost tangent to the production curves. (D) Recovery time as a function of the resilience parameter *R*. When *R* is large, recovery takes on the order of days. Below *R* = 1/3, recovery slows significantly (ACSD), and the trajectory is non-exponential, with a pronounced plateau at high symptoms. Recovery time diverges below *R* = 1/4, indicating bistability and the potential for chronic PTSD.

We model trauma as an initial condition of high *x*. Those who are not susceptible to PTSD (*R* > 1/4) recover toward the healthy state after the trauma. The recovery timescale (time to return halfway to the healthy state) depends on the value of R (Figure 2D).

In order to discuss time in real units, we need to determine the threshold between AcSD and resilience. We set this threshold to the parameter value at which the trajectory of *x* first has an inflection point, namely *R* = 1/3. The inflection point can be seen from the analysis of the second derivative (see STAR Methods). Based on the DSM-5 definition of PTSD, which requires symptoms to persist for at least one month post-trauma, we normalize time such that the recovery time at *R* = 1/3 is set to one month, resulting in dimensionless time *τ* = 1/4 corresponding to one week.

For large *R*, symptoms decline exponentially with time (Figure 2B, curve labeled resilient). For *R* values lower than 1/3, the recovery time rises rapidly as *R* is lowered, and reaches months. In this range, recovery curves have a plateau of moderate to high symptoms that lasts for months to years and then recovers (Figure 2B, curve labels AcSD for AcSD). The recovery time diverges at *R* = 1/4 when symptoms shift to the PTSD state indefinitely (Figure 2B curve labeled PTSD). The exact relation between *R* and recovery time is shown in Figure 2D. Near the critical value, the recovery time diverges as $\tau_{recovery}\propto \frac{1}{\sqrt{R-1/4}}$.

These dynamical trajectories are in qualitative agreement with observed longitudinal symptoms of patients after a traumatic event (Figure 2A).^25^ A detailed quantitative fitting of the model to a wide range of published trajectories is presented in a dedicated section later in discussion.

The reason for the slow recovery times of months is known in dynamical systems theory as a “ghost phenomenon.” When R is only slightly larger than the bifurcation point *R* = 1/4, the removal and production curves nearly cross (Figure 2C). The rate of change of *x* is thus very slow and diverges at *R* = 1/4, where the curves are tangent.

### A distribution of the resilience parameter in the population can explain susceptibility to PTSD

**Figure 3:**
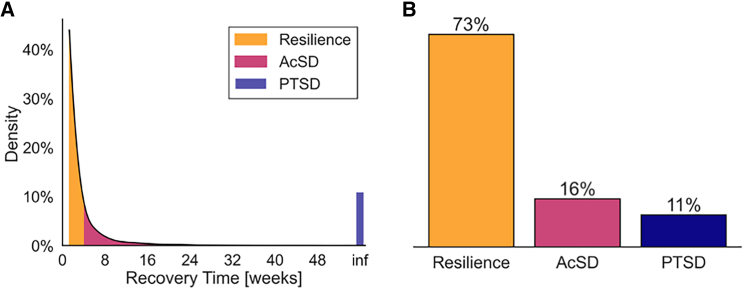
Model-predicted distribution of recovery outcomes following trauma with variation in the parameter *R* (A) Simulated recovery times, based on a population-wide distribution of the resilience parameter R, show a broad range among non-susceptible individuals (resilience, orange; acute stress disorder, pink), and chronic symptoms for susceptible individuals (PTSD: blue). (B) These simulations predict a population-level distribution consistent with clinical data: ∼11% develop PTSD, ∼16% show prolonged but reversible symptoms (AcSD), and ∼73% recover rapidly.

To further explore susceptibility when the parameter *R* varies in the population, we model *R* as a polygenic trait (with multiplicative effects) that follows a log-normal distribution (a normal distribution gives similar results). We find that a distribution with mean *R* = 0.5 and standard deviation *SD*(*R*) = 0.25 produces a range of recovery times close to that observed (Figure 3A). Defining resilience as *R* > 1/3, AcSD as 1/4 < *R* < 1/3, and PTSD as *R* < 1/4 (bistability), the model predicts a distribution of phenotypes following a traumatic event of about 75% resilient, 15% AcSD, and 10% PTSD (Figure 3B).

We compared this to observed distributions of trauma responses, using the review of Galatzer-Levy et al.^5^ which collected prevalence in diverse types of trauma (Table 1). The PTSD prevalence in many kinds of trauma (military, accident, life events, health events) is around 10–20%, AcSD is 15–25%, and resilience is 62–78%, in rough agreement with the model.

**Table 1:** Prevalence of PTSD symptom trajectories across different trauma types based on Galatzer-Levy et al.^5^

PTE type	Chronic (mean ± SE)	Acute stress disorder (mean ± SE)	Resilient (mean ± SE)
Military	9% ± 3%	13% ± 4%	78% ± 4%
Civilian trauma/accident	12% ± 2%	24% ± 4%	62% ± 3%
Refugee/War	24% ± 1%	76% ± 1%	–
Loss	12% ± 2%	15% ± 4%	64% ± 3%
Major life events	12% ± 3%	4% ± 1%	74% ± 4%
Children	3% ± 1%	32% ± 13%	52% ± 10%
Health events	14% ± 5%	18% ± 8%	65% ± 7%

Two trauma types have different prevalence profiles: Childhood trauma has less PTSD (3%), and war/refugee trauma has more (24%). These are complex traumas with potentially many traumatic events and may require modification to the model.

We conclude that genetic and environmental variability in the parameter *R* between individuals can provide resilience, AcSD, and PTSD prevalence in many (but not all) types of trauma.

### Repeated sub-threshold traumatic events can cause PTSD

To investigate polytrauma, in which multiple stressors occur in a relatively short period of time, we simulated the model with an external stress input u. This stress pushes *x* to higher values, namely $\frac{dx}{dt}=u+x^{2}\left(1-x\right)-Rx$.

**Figure 4:**
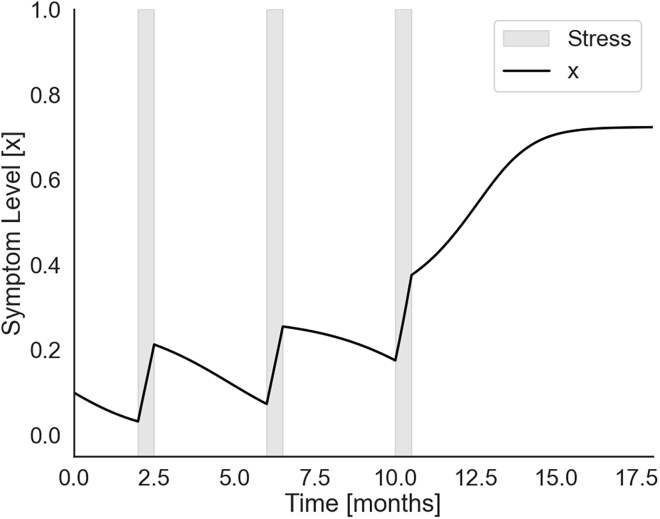
Cumulative exposure to sub-threshold traumas can cause PTSD Simulation of the model (*R* = 0.2, bistable regime) subjected to three repeated stress sessions (gray bars, stress magnitude *u* = 0.1, duration 0.5 months) separated by intervals of 4 months. While a single stressor of this magnitude allows for recovery to the healthy state, the proximity of repeated stressors causes symptom accumulation. The third stressor event pushes the symptom level *x* beyond the unstable fixed point, causing a transition into the chronic high-symptom PTSD state even after the stressor is removed. This demonstrates how polytrauma or cumulative adversity can generate PTSD in susceptible individuals even if a single event would not.

We introduce a stress event of two weeks (*u* = 0.1) which in itself does not cause PTSD in a susceptible individual (*R* = 0.2). However, three such events spaced by four months combine to cross the unstable fixed point and lead to chronic PTSD symptoms (Figure 4). In general, stressors that occur closer together than the time for symptoms to strongly decay (on the order of six months) can combine to cause PTSD even if each stressor on its own would not.

### Hysteresis indicates that the PTSD state is easier to enter than to exit

We can also use the model to ask questions about the response to treatment. We model treatment as a force (negative *u*) pushing *x* down toward the healthy state, in contrast to trauma modeled as above with a positive *u*.

We plot the steady-state symptom load x_st_ as a function of a constant level of *u* for a susceptible individual (*R* = 0.2). Starting at zero symptoms, *u* need to surpass a critical value *u* = 0.25 in order to induce PTSD.

**Figure 5:**
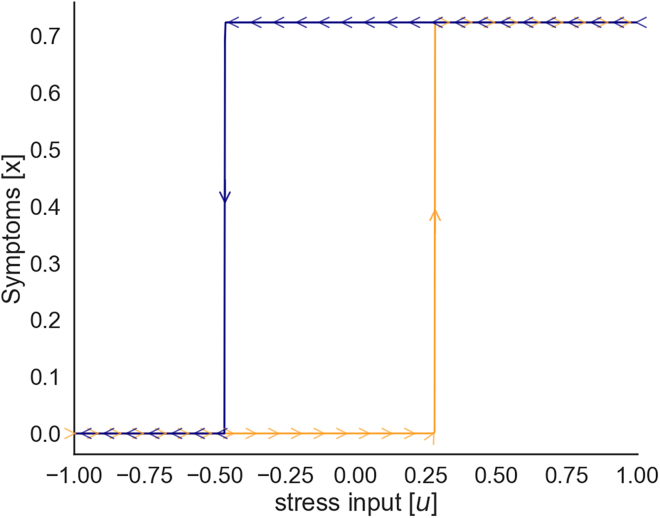
The PTSD state is easier to enter than to exit, as evident in a hysteresis curve Setting *R* = 0.2 and starting from a healthy state (orange curve), symptoms remain low until stress input exceeds a threshold (∼0.25), triggering a jump to high symptoms. Starting from the PTSD state (blue curve), symptoms stay high unless treatment (reduction in stress input) is strong enough (below ∼ -0.5) to achieve recovery.

Next, we consider therapy by starting at the high symptom state and applying different values of negative *u*. The steady-state symptoms now remain high unless *u* is below a second threshold, which is lower in absolute value than the first, *u* = −0.5 (Figure 5). This is known as *hysteresis*, a feature of many bistable systems. In a certain sense, PTSD is easier to enter than to exit. The reason is the self-catalyzing term in which *x* enhances its own production. This causes a barrier to treatment at high symptom levels.

### Treatment is predicted to show a threshold effect

**Figure 6:**
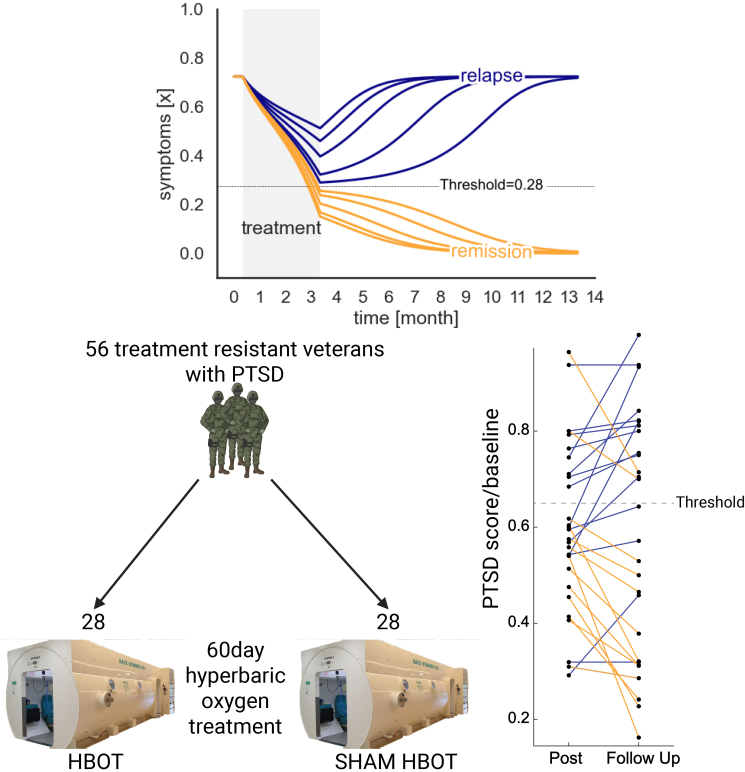
Treatment threshold effect - improvement smaller than a threshold leads to relapse, and beyond it to further recovery (A) Dynamics of the model with varying treatment strengths (*u*) over 3 months. Treatment that leads to improvement of less than a threshold at the end of treatment is followed by worsening of symptoms, whereas improvement above the threshold leads to further recovery. Here *R* = 0.2. (B) Sham controlled double blind experiment on treatment-resistant veterans with PTSD.^17^^,^^18^ (C) Normalized CAPS scores immediately after 3 months of hyperbaric oxygen therapy (HBOT) and in a 3-month follow-up, normalized to pretreatment baseline, replotted. The threshold of 35% separates mild improvement that tends to relapse (blue) and substantial improvement that shows further recovery (yellow).

We also explored the symptom dynamics following different strengths of treatment. Weak treatment (|*u*|<0.055 for 90 days) causes a temporary reduction in symptoms that increase after treatment and return to the PTSD fixed point on a timescale of months. Strong treatment (|*u*|>0.055 for 90 days) results in further improvement after treatment ceases (Figure 6A).

The model thus predicts a threshold effect for treatment. Treatment that ends with symptoms above the unstable fixed point will return to PTSD, whereas treatment that ends with symptoms below the fixed point will lead to further healing.

We compare this analysis to data from a biological treatment modality for PTSD: 60 days of HBOT.^17^^,^^18^ The dataset includes 28 treatment-resistant veterans who received active treatment as part of the intervention arm in a sham-controlled, double-blind clinical trial. Participants in the sham group (*n* = 28) did not show improvement.^17^^,^^29^ All participants were scored for symptoms before, right after, and three months after treatment, by means of the CAPS for DSM-5 symptoms score scale.

Participants who experienced a substantial improvement in their CAPS score immediately after treatment, exceeding a threshold of approximately 35%, tended to continue improving in the 3-month follow-up. In contrast, those who showed milder improvement, below 35% after treatment, tended to lose these improvements in the three-month follow-up. This aligns with the model’s prediction of a treatment threshold: only interventions that reduce symptoms below the unstable fixed point lead to sustained recovery, while insufficient improvement results in relapse to the high-symptom PTSD state.

The model also suggests a timescale for treatment and post-treatment dynamics of several months, which aligns with observations.

We conclude that the model predicts a *treatment threshold* phenomenon - treatment that causes improvement beyond a threshold leads to further recovery. In contrast, treatment that fails to achieve this threshold results in a return to the high symptom state.

### The model captures the trajectories of post-trauma symptoms across datasets

**Figure 7:**
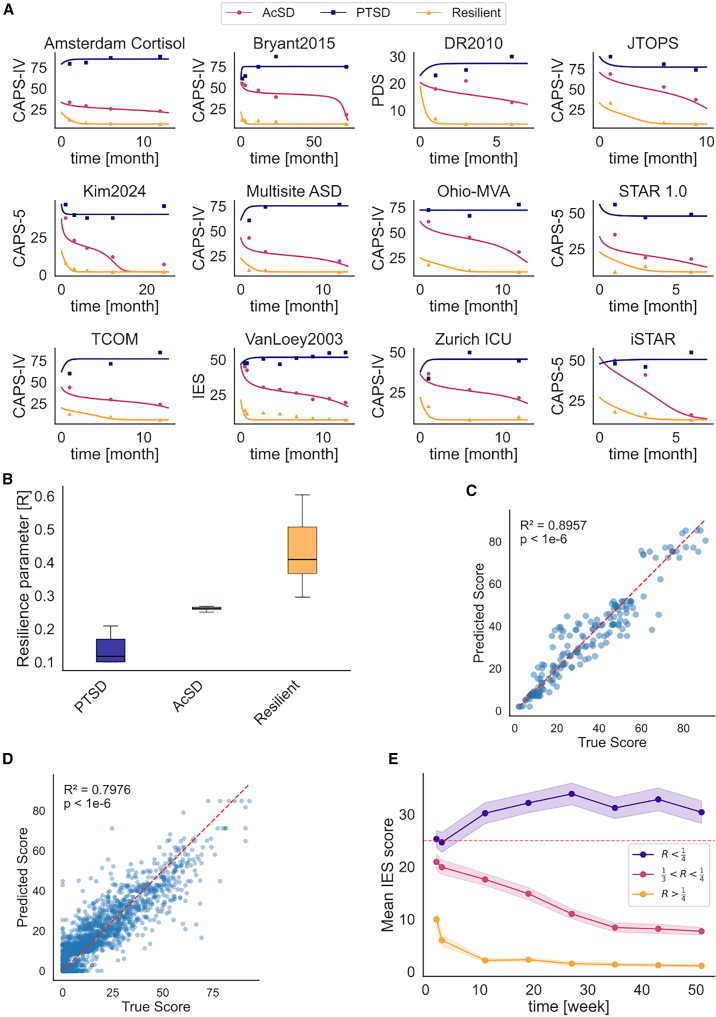
Model validation across cohort-level datasets In panels A, C, and D, R^2^ is the coefficient of determination and reported *p*-values are from a two sided Pearson correleation between observed and model-predicted symptom scores (*p* < 1e-6 in all cases). (A) Cohort-level longitudinal trajectories from published trauma studies, grouped into resilient, AcSD, and chronic PTSD courses (resilience: orange; AcSD: pink; PTSD: blue); solid lines are model fits. (B) Fitted resilience parameter *R* for each trajectory type. Boxes represent the upper and lower quartiles, and whiskers denote 1.5 times the interquartile distance (IQR). Boundaries follow the model predictions: PTSD *R*<1/4; AcSD 1/4 < *R* < 1/3; Resilient *R* > 1/3. (C) Comparison of the empirically observed symptom scores (True Score, x axis) with the values predicted by the minimal dynamical model (Predicted Score, y axis) for all time points across the grouped datasets. The red dashed line represents a perfect prediction (y = x). The model achieves *R*^2^ = 0.9 showing strong agreement. (D) Comparison of the model’s performance on individual longitudinal data from the Van Loey et al.^32^ cohort. The model achieves *R*^2^ = 0.8, again showing strong agreement. (E) Average symptom trajectories (Mean ± SEM) according to the best fit *R* values reveal three distinct temporal patterns: Chronic PTSD (Purple, *R* < 1/4): Individuals falling into the model’s bistable regime show persistently elevated symptoms that remain above the clinical cutoff (red dashed line, IES score = 37). Acute Stress Disorder (Pink, 1/4 < *R* < 1/3) and Resilience (Orange *R* > 1/3).

We compared the model predictions to cohort-level longitudinal symptom data from 12 published studies spanning 7 months to 2 years post-trauma (Figure 7A). For each dataset, we fitted the model to the trajectories corresponding to chronic, slow-recovery, and resilient courses of post-trauma symptoms.

Since the data are in terms of symptom scale scores (such as CAPS-4,^33^ CAPS-5,^1^ IES^34^), we convert the variable *x* to a symptom score by multiplying *x* by a scale factor w that is fit for each trajectory. This scale factor is the score at *x* = 1. Thus, for each trajectory, we fit the resilience parameter *R* and the scale parameter *w*, and assume for all fits that dimensionless time *τ* = 1/4 corresponds to one week. The model reproduced these trajectories very well (Rˆ2 = 0.9, *p* < 0.0001, Figure 7A).

The fitted resilience parameter *R* values were low (*R* < 1/4) for chronic trajectories, intermediate (1/4 < *R* < 1/3) for slow-recovery trajectories, and high *R* (*R* > 1/3) for resilient trajectories (Figure 7B). This separation emerged without imposing explicit constraints on R, indicating that the model intrinsically captures the underlying dynamical structure of symptom evolution.

We also fit 270 individual symptom trajectories from Van Loey et al.,^32^ and found a good fit (*R*^2^ = 0.8, *p* < 0.0001, Figure 7D). Again, values of *R* below 0.25 corresponded to chronic trajectories, those with *R* > 1/3 corresponded to low symptoms and resilience, and intermediate *R* showed initial high symptoms that reduced over time (Figure 7E). We conclude that the present model captures the dynamics of symptom time courses in resilience, AcSD, and PTSD.

## Discussion

We presented a minimal model for the bistability, timescales, and susceptibility of PTSD. The model variable is symptom load that is self-enhancing and undergoes removal. A single dimensionless parameter, the ratio of removal to production rates, determines whether an individual is susceptible to PTSD. The model captures the temporal trajectories of symptoms in multiple types of trauma when compared to published studies. The model predicts that treatment that achieves improvement beyond a threshold will show further decrease in symptoms, whereas treatment that does not achieve this threshold will relapse to the PTSD state. This agrees with observations on hyperbaric oxygen treatment. The slow timescale of months that characterizes recovery in individuals with acute stress disorder is suggested to occur due to a dynamical ghost where removal and production are nearly balanced.

Susceptibility in the model is governed by a dimensionless ratio of removal and production parameter (*R* = *b*/*ac*). This “resilience parameter” *R* is assumed to vary in the population due to genetics and early life experience. Those with low *R* (low removal rate compared to self-enhancement time carrying capacity), namely *R* < 1/4, are susceptible to PTSD and have the potential for bistability. Those with *R* > 1/4 are resilient, although individuals close to the threshold *R* = 1/4 will experience a very long recovery time, scaling as $\frac{\pi }{\sqrt{R-1/4}}$. A polygenic-like distribution of *R* captures the prevalence of PTSD, AcSD, and resilience in many (but not all) trauma types.

The model predicts a treatment threshold effect, closely linked with bistability. It predicts a threshold in agreement with clinical observation on hyperbaric oxygen treatment. A similar effect with a 30–35% threshold was also reported for other PTSD treatment modalities,^19^^,^^35^^,^^36^ and 30–35% improvement is often used as a criterion for successful treatment in the PTSD field. The model also captures the timescale required for treatment, and offers guidelines for expected post-treatment dynamics that can help plan the timing of patient follow-up.

The model may also relate to late-onset PTSD, which can occur months to years after the original trauma.^4^^,^^37^^,^^38^ One possibility is that the trauma trigger only crosses the threshold at a late time for psychosocial reasons. Another possibility is that late-onset PTSD occurs when the parameter *R* declines over time due to aging, stress, or other factors. An initially resilient individual can become susceptible, and then a new trigger (or possibly a memory of a former trigger) can induce PTSD. A third possibility is dynamical. It applies to cases where symptoms are moderate for months before crossing into PTSD (see SI2). Future studies can help test these possibilities.

It would be of interest to find biological correlates for the resilience parameter *R*. An analogous dynamic resilience index has been recently derived from longitudinal symptom and neuroimaging data.^39^ Whole-brain activity analysis indicated that resilience is promoted by the activation of regions involved in higher-level cognitive functioning, reward valuation, and salience detection in response to reward, whereas resilience is hampered by posterior default mode network activation to threat and reward. This supports the feasibility of low-dimensional resilience measures.

Possible drivers of x may lie in the fronto-limbic circuitry that includes the amygdala and the hippocampus. Amygdala activity may drive x because of its self-enhancing dynamics and hyperactivity in PTSD.^13^^,^^40^^,^^41^ The removal term may be due to hippocampal or prefrontal inputs that inhibit the amygdala.^41^^,^^42^ Thus, one possibility for the parameter *R* is the ratio of amygdala self-enhancement strength and hippocampal/PFC inhibition strength on the amygdala. Future longitudinal studies with brain imaging, using therapy as a perturbation, might help to test such hypotheses.

Increasing the resilience parameter R provides a potential therapeutic target. Reducing self-enhancement or increasing the removal of x can both promote resilience. By raising R beyond R_c_ = ¼, one can eliminate the PTSD fixed point and force the dynamics to return to health as the only fixed point option. Such a strategy was recently found beneficial in fibrosis, a condition of excess scarring. A minimal mathematical model indicated that the fibrosis fixed point can be eliminated by reducing a certain autocrine loop.^43^ Reducing this autocrine loop experimentally prevented and reversed fibrosis in the hearts and livers of mice.^28^^,^^44^^,^^45^

The model can be used to simulate and rationalize treatment schedules. Different modalities of treatment duration and cessation can be readily simulated, with longer treatment indicated for individuals with low R. Our results suggest that treatment should not be stopped until a threshold improvement is reached. Likewise, continuing treatment long after this threshold is crossed may be unnecessary since dynamics will now lead to healing. The timescale of months in the model agrees with the minimal three-month treatment duration that seems to be effective for PTSD across treatment modalities.^46^^,^^47^^,^^48^

This bistable model for PTSD adds to recent mathematical models for major depression and bipolar disorder.^24^^,^^49^ Both models were based on the HPA axis and had timescales of months. The major depression model was based on bistability due to a toggle-switch-like circuit where cortisol inhibits the hippocampal inhibition of the HPA axis. The bipolar disorder model was based on cortisol fluctuations over months due to noisy oscillations in the HPA axis that trigger cortisol-induced mania in susceptible individuals. It would be interesting to connect these models in order to study comorbidity with depression, which is often observed in PTSD.

This study may offer a foundation for future studies that might have clinical relevance. The parameter R for an individual can be inferred from symptom time courses, as shown in Figure 7. Thus, future studies might investigate genetic and environmental factors that affect or predict *R*. Future studies on diagnosis or intervention might stratify patients by *R*. For example, a mathematical model of diabetes helped define insulin resistance as a key parameter, leading to future studies providing clinically actionable measurements of insulin resistance.^50^^,^^51^^,^^52^

### Limitations of the study

This study is the initial step of the development of a model for trauma dynamics and timescales. Limitations of this study include the deliberate simplicity of the model that misses the complexity of PTSD. Future improvements can address the way PTSD outcomes are affected by pre-trauma PTSD levels, severity, and nature of the trauma or polytrauma. Resilience can change over time and in different states, as perhaps occurs in delayed onset PTSD. In addition, future work can address the complexity of how adversity impacts symptom trajectories after the trauma, including that caused by the symptoms themselves.

Future work must also rigorously test the model against out-of-sample empirical datasets, utilizing formal fitting procedures and robust prediction accuracy assessments to validate its clinical utility.

## Resource availability

### Lead contact

Further information and requests for resources and reagents should be directed to and will be fulfilled by the lead contact, Uri Alon (uri.alon@weizmann.ac.il).

### Materials availability

This study did not generate new unique reagents.

### Data and code availability

• Data: All data generated in this study are included in the article. This paper analyzes existing datasets that are publicly available and referenced in the key resources table.
• Code: All original code has been deposited at Zenodo and is publicly available as of the date of publication. DOIs are listed in the key resources table.
• Additional information: Any additional information required to reanalyze the data reported in this paper is available from the lead contact upon request.

## Acknowledgments

We thank all members of the Alon Lab for discussions. This work was supported by the European Research Council (ERC; Grant Agreement No. 856487), the Sagol Institute for Longevity Research at the Weizmann Institute of Science, and the Weizmann–Knell Family Institute for Artificial Intelligence. U.A. is the incumbent of the Abisch-Frenkel Professional Chair.

## Author contributions

Y.G.: conceptualization, software, formal analysis, validation, investigation, visualization, methodology, writing – original draft, and writing—review and editing. D.D.: conceptualization, resources, data curation, investigation, and writing—review and editing. K.D.-B.: conceptualization, investigation, resources, and data curation. A.M.: supervision, investigation, methodology, and writing—review and editing. U.A.: conceptualization, supervision, funding acquisition, investigation, writing – original draft, and writing—review and editing. All co-authors have read and approved the final version of the manuscript.

## Declaration of interests

The authors declare no competing interests.

## STAR★Methods

### Key resources table

**Table:** 

REAGENT or RESOURCE	SOURCE	IDENTIFIER
**Deposited data**		

Minimal PTSD Model Code	This paper	Zenodo: https://doi.org/10.5281/zenodo.19736998
International Consortium to Predict PTSD (ICPP) dataset	Qi et al.^53^	https://doi.org/10.1080/20008198.2018.1476442
Multisite Acute Stress Disorder dataset	Bryant et al.^54^	https://doi.org/10.4088/jcp.v69n0606
Jerusalem Trauma Outreach and Prevention Study (JTOPS) dataset	Shalev et al.^55^	https://doi.org/10.1001/archgenpsychiatry.2011.127
Tachikawa Cohort of Motor Vehicle Accidents (TCOM) dataset	Matsuoka et al.^56^	https://doi.org/10.1007/s00127-008-0438-6
Ohio Motor Vehicle Accident (Ohio-MVA) dataset	Delahanty et al.^57^	https://doi.org/10.1016/s0887-6185(02)00185-8
Zurich Intensive Care Unit dataset	Hepp et al.^58^	https://doi.org/10.1192/bjp.bp.106.030569
Amsterdam cortisol dataset	Mouthaan et al.^59^	https://doi.org/10.1016/j.psyneuen.2014.04.001
Burn injury cohort dataset	Van Loey et al.^32^	https://doi.org/10.1023/A:1024465902416
Injury cohort (6-year) dataset	Bryant et al.^60^	https://doi.org/10.1192/bjp.bp.114.145516
STAR 1.0 dataset	Tomas et al.^61^	https://doi.org/10.1002/jts.22868
iSTAR dataset	Tomas et al.^61^	https://doi.org/10.1002/jts.22868
Physical injury cohort dataset	Kim et al.^25^	https://doi.org/10.1155/2024/5570405

**Software and algorithms**		

Python (v3.13)	Python Software Foundation	https://www.python.org; RRID:SCR_008394
NumPy (v2.3.4)	Harris et al.^62^	https://numpy.org; RRID:SCR_008633
SciPy (v1.16.3)	Virtanen et al.^63^	https://scipy.org; RRID:SCR_008058
Pandas (v2.3.3)	McKinney.^64^	https://pandas.pydata.org; RRID:SCR_018214
Matplotlib (v3.10.7)	Hunter.^65^	https://matplotlib.org; RRID:SCR_008624
Seaborn (v0.13.2)	Waskom.^66^	https://seaborn.pydata.org; RRID:SCR_018132
matplotlib-label-lines (v0.8.1)	Cadiou.^67^	https://github.com/cphyc/matplotlib-label-lines

### Experimental model and study participant details

No new experimental models or participants were recruited.

### Method details

#### Fixed points

Consider the dimensionless equation for PTSD symptoms dynamics:

$$\frac{dx}{dt}=x^{2}\left(1-x\right)-Rx$$

Fixed points occur when dx/dt = 0. The fixed point *x* = 0 exists for all values if R and corresponds to the healthy state, and is stable since the linearized solution has a negative eigenvalue -R. The other fixed points obey *x*^2^-*x*+*R* = 0. The discriminant *D* = 1-4*R* determines the nature of the remaining fixed points. For $R<\frac{1}{4}$, there are two distinct real solutions, indicating a total of three fixed points including the one at zero. For $R=\frac{1}{4}$, the two solutions merge into one, indicating a bifurcation point. This bifurcation point is half-stable: stable from the right and unstable from the left. For $R<\frac{1}{4}$, no real positive fixed points exist apart from x = 0. Thus, $R=\frac{1}{4}$ marks the bifurcation point.

#### Estimating recovery time near the dynamical ghost

The model shows a saddle node bifurcation and a square-root scaling law. Near the bifurcation, the time spent near the “ghost” is dominated by the distance *r* of *R* from the bifurcation point and scales $T_{ghost}=\frac{\pi }{\sqrt{r}}$. To approximate *r*, we first note that at *R* = 1/4, the rate function $\frac{dx}{dt}$ achieves its maximum at *x* = 1/2. Evaluating the distance of $\frac{dx}{dt}$ at *x* = 1/2 as a function of *R*

$$\frac{dx}{dt}{|}_{x=0.5}=0.5\left(0.25-R\right)$$

Because this is negative for *R* > 0.25, the distance is *r* = 0.5(*R*-0.25). Thus

$$T_{ghost}=\frac{\sqrt{2}\pi }{\sqrt{R-1/4}}$$

#### *R* = 1/3 as a transition to a recovery plateau

Beyond the critical bifurcation at *R* = 1/4, an additional qualitative change occurs near *R* = 1/3, where the local minimum and maximum of $\frac{dx}{d\tau }$ collide and annihilate. This causes the rate of x to decrease monotonically, eliminating intermediate slowing phases. To analyze this, we first derive the extrema of $\frac{dx}{d\tau }$, namely $\frac{d^{2}x}{d\tau^{2}}=2x-3x^{2}-R=0$, obtaining $x_{critical}=\frac{1\pm \sqrt{1-3R}}{3}$. When *R* > 1/3, the discriminant is negative and no real solutions exist, implying that $\frac{dx}{dt}$ is strictly monotonic, and the symptoms recover without a slowing phase. When *R* < 1/3, the discriminant is positive, resulting in two real solutions corresponding to a local maximum and a local minimum of $\frac{dx}{dt}$. Thus, *R* = 1/3 marks a threshold between simple monotonic recovery and the appearance of a recovery plateau.

#### Simulation of treatment and polytrauma

Traumatic stress was modeled as a positive additive input *u*(*t*) > 0 in the dimensionless model, and treatment was modeled similarly with a negative *u*(*t*) < 0

$$\frac{dx}{dt}={u+x}^{2}\left(1-x\right)-Rx$$

#### Numerical simulations and data analysis

The model consists of a non-linear ordinary differential equation (Eq. 2). To generate the time-course trajectories and hysteresis curves, the equation was solved numerically using Python (version 3.13) with the scipy.integrate.solve_ivp solver.

#### Data sources

For the cohort-level trajectories, data were drawn from multiple longitudinal cohorts. We used cohort-level trajectories exactly as identified in the source studies and harmonized class names across datasets to ensure comparability. The following contributing studies were taken from the review of Lowe et al.^27^ The International Consortium to Predict PTSD (ICPP),^53^ The Multisite Acute Stress Disorder study (Multisite ASD),^54^ Jerusalem Trauma Outreach and Prevent Study (JTOPS),^55^ Tachikawa Cohort of Motor Vehicle Accidents (TCOM),^56^ Ohio Motor Vehicle Accident study (Ohio-MVA),^57^ Zurich Intensive Care Unit study (Zurich ICU),^58^ and the Amsterdam Cortisol study.^59^ Additional harmonized datasets included the Burn Injury Cohort,^32^ the six-year longitudinal injury cohort^60^ the International Consortium to Predict PTSD,^53^ and data from Tomas et al.,^61^ STAR 1.0 (Study on Trauma and Resilience) described in Fitzgerald et al.,^68^ Geier et al.,^69^ and Weis et al.,^70^ and iSTAR (Imaging Study on Trauma and Resilience), detailed in Bird et al.,^71^ Webb et al.,^72^ and Weis et al.,^73^ which identify distinct posttraumatic stress symptom trajectories. Finally, we included data from Kim et al.^25^ on hospitalized patients with physical injuries. Together, these cohorts provide a comprehensive, multinational resource for modeling heterogeneity in posttraumatic stress responses over time.

#### Model fitting and parameter estimation

##### Data processing

Longitudinal symptom data were processed to extract individual trajectories. We included individuals who had valid symptom measurements at three or more time points to ensure sufficient data for parameter estimation. Measurement times were mapped to weeks post-trauma (Weeks 2, 3, 11, 19, 27, 35, 43, and 51) to align with the data collection schedule.

### Quantification and statistical analysis

We fitted the dynamical model $\frac{dx}{dt}=x^{2}\left(1-x\right)-Rx$ to the observed trajectories. To map the dimensionless model variable *x* (bounded between 0 and 1) to the clinical symptom scale, we introduced a scaling parameter *w*_*i*_. The predicted symptom score *y*_*pred*_(*t*) was defined as:

$$y_{pred}\left(t\right)=w_{i}\cdot x\left(t\right)$$

Where *x*(*t*) is the numerical solution of the differential equation.

#### Optimization procedure

For each individual, we estimated three parameters simultaneously: the resilience parameter *R*, the initial symptom state *x*_0_, and the scaling factor *w*_*i*_. We used the Differential Evolution algorithm, a stochastic global optimization method, to minimize the Sum of Squared Errors (SSE) between the model prediction and the observed data:

$$SSE=\sum_{i=1}^{N}{\left(y_{obs}\left(t_{i}\right)-w_{i}\cdot x\left(t_{i}\right)\right)}^{2}$$

To ensure the model remained grounded in clinical and physiological reality, the optimization procedure was strictly constrained to biologically and clinically plausible ranges. The resilience parameter *R* was bounded between 0.1 and 1.0. The initial symptom state x_0_ was restricted to values between 0.7 and 1.0, a range that reflects the characteristic high symptom load typically observed during the acute phase immediately following a traumatic event. Finally, the scaling factor w_i_ was bounded between 20 and 100 to properly accommodate the different standard ranges used in clinical symptom severity scales across the various datasets. Goodness of fit was assessed using the coefficient of determination R^2^ and associated *p*-values.
