# Supplementary material for: A minimal model of PTSD susceptibility and timescales
Source: iScience. 2026 Jun 24;29(7):116391. doi: 10.1016/j.isci.2026.116391 (PMC13377864; doi:10.1016/j.isci.2026.116391)
Supplement: Document S1. Figures S1–S3 and Methods S1 and S2 [file mmc1.pdf]

iScience, Volume *29*

Supplemental information

A minimal model of PTSD susceptibility and timescales

Yaniv Grosskopf, Dor Danan, Avi Mayo, Keren Doenyas-Barak, and Uri Alon

Supplemental information

Index

Method S1: An alternative threshold-based formulation of the bistable model yields equivalent recovery dynamics and treatment thresholds, related to Figure 1.
Method S2: Delayed-onset PTSD can be explained by time-varying resilience, secondary triggers, or a dynamical latency near the unstable fixed point, related to Discussion.

Method S1: An alternative threshold-based formulation of the bistable model yields equivalent recovery dynamics and treatment thresholds, related to Figure 1.

We explored whether the observed bistability in our PTSD model is sensitive to the specific functional form of the production term. An alternative approach to modeling trauma response involves a threshold-based model, where symptom self-enhancement is triggered when symptom load *x* exceeds a threshold x_T. This formulation also generates bistability and produces qualitative results consistent with our primary model, including distinct recovery trajectories and susceptibility regimes. Formally, the model takes the form:

$$\frac{dx}{dt} = ax \cdot \Theta(x) - rx$$

Where Θ is a step function, defined as

$$\Theta(x) = \begin{cases} 1, & 0 \le x \le 1 \\ 0, & else \end{cases}$$

The bifurcation in this model occurs when $r = a$. To make the comparison to our primary model straightforward, we set $a = \frac{1}{4}$, thus the bifurcation happens at the same r value as the original cubic model. Based on the results from Danan et al.[29], we use a threshold value of 0.65. This implies that recovery occurs only after symptoms are lowered by 35%.

The results presented below are derived using these specific parameters. The advantage of this model over the one in the main text is that the threshold for treatment is independent of R in susceptible individuals, consistent with the results of Danan et al. [29]

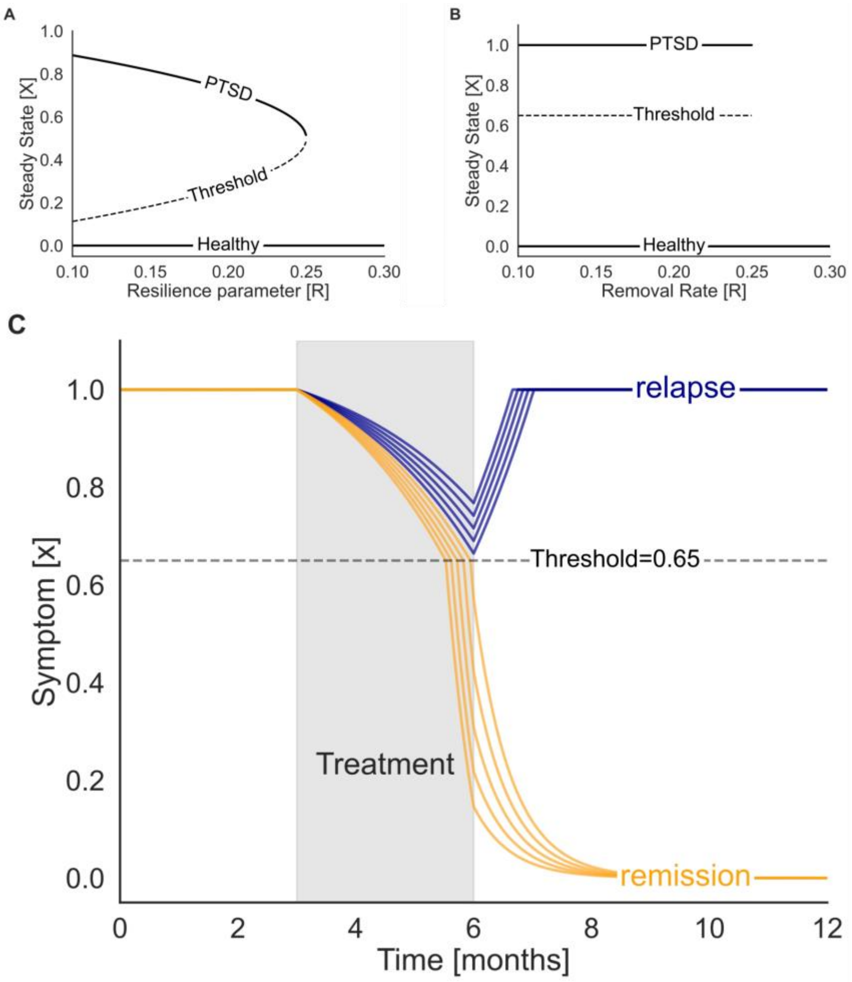

Figure S1. Bifurcation comparison and dynamics of treatment in the threshold model, related to Figure 1.
(A) Bifurcation diagram of the cubic model steady states as a function of the resilience parameter (*R*), as in Figure 1C. In this formulation, the critical threshold for recovery is dependent on *R*. (B) Bifurcation diagram of the alternative threshold model, demonstrating that the critical threshold is independent of the parameter R. (C) Simulation of treatment trajectories using the threshold model, confirming the presence of a fixed critical threshold for remission (at $x = 0.65$).

Method S2: Delayed-onset PTSD can be explained by time-varying resilience, secondary triggers, or a dynamical latency near the unstable fixed point, related to Discussion.

Delayed onset PTSD occurs when individuals do not meet full diagnostic criteria immediately after a traumatic event but develop the disorder months or even years later. One possibility is that resilience *R* drops over time due to biological factors such as aging, or environmental change and adversity. Another possibility is that the traumatic event does not cross the threshold, and a healthy state is resumed, but a second triggering event causes intrusive symptoms related to the original trauma that cross the threshold long after the trauma.

A third possibility is dynamical. It applies to cases where symptoms are moderate for a long time before crossing into PTSD. This can be explained by the model's dynamics near the unstable fixed point. In the bistable regime ($R < 1/4$), the healthy state and the PTSD state are separated by an unstable fixed point, x_u, which acts as a threshold. When a traumatic event induces an initial symptom load, x_0, that only marginally exceeds this threshold ($x_0 = x_u + \epsilon$), the system enters a region where the rate of change $\frac{dx}{dt}$ is positive but small.

Because the production and removal rates are nearly balanced at this point, the symptom trajectory "hovers" near the threshold for an extended period, creating a latency phase where the individual exhibits sub-syndromal symptoms. Over time, the state slowly drifts away from the unstable fixed point, and the self-enhancing production term increasingly dominates, causing an acceleration in symptom accumulation that manifests clinically as the late emergence of the full PTSD phenotype.

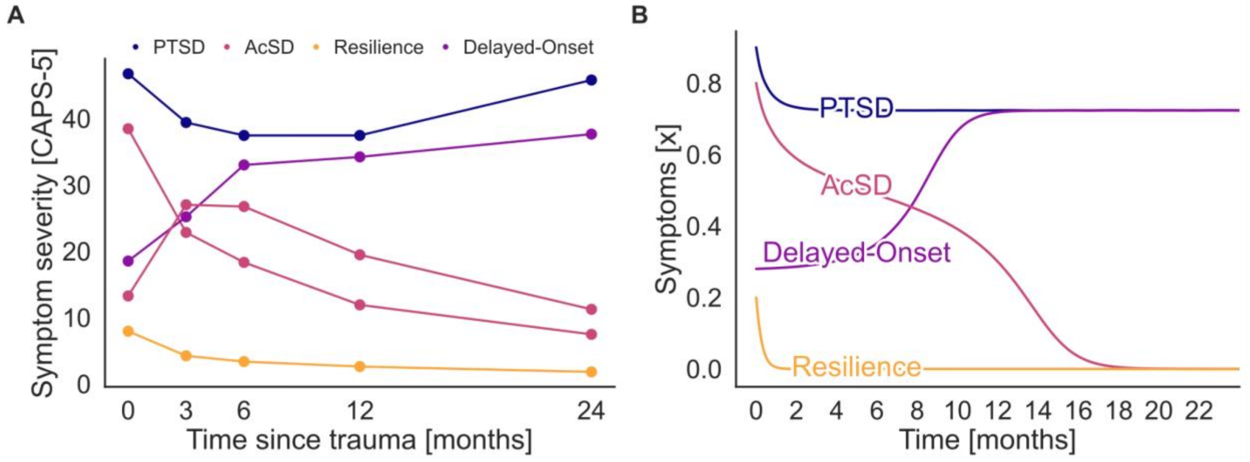

Figure S2. Comparison of observed clinical trajectories and model simulations, related to Figure 7. (A) Empirical symptom trajectories (CAPS-5) over 24 months showing four distinct clinical phenotypes: Chronic PTSD, Acute Stress Disorder (AcSD), Resilience, and Delayed-Onset. (B) Model simulations reproducing these four characteristic dynamic behaviors.

The model fits well to delayed-onset PTSD trajectories (purple lines in panels in Fig. S2). It shows $R^2 = 0.81$.

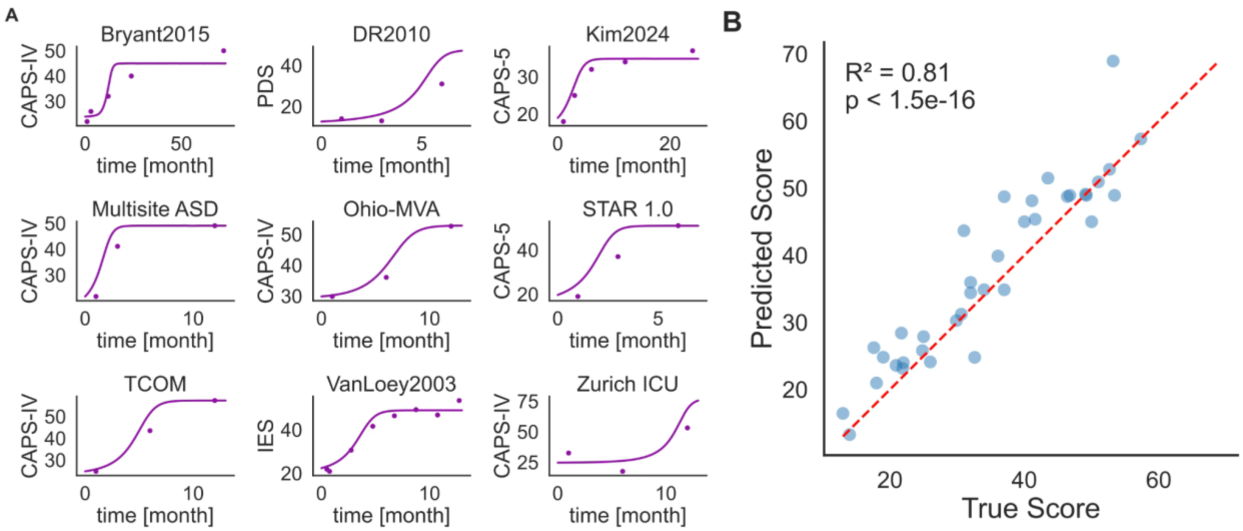

Figure S3. Quantitative validation of model fits to empirical delayed-onset trajectories, related to Figure 7. (A) Model fits to longitudinal data from nine independent studies reporting delayed-onset PTSD symptoms. (B) Comparison of the model predicted symptom scores to the actual observed scores across all datasets with delayed onset trajectory. The red dashed line represents the identity line (y=x), indicating perfect prediction.
